# Supplementary material for: Nationwide assessment of leadership development for graduate students in the agricultural plant sciences
Source: PLoS One. 2023 Apr 17;18(4):e0279216. doi: 10.1371/journal.pone.0279216 (PMC10109473; doi:10.1371/journal.pone.0279216)
Supplement: S1 Table — (DOCX) [file pone.0279216.s001.docx]

**Supporting information**

**S1 Table. Themes and their definitions developed based on patterns observed in the text responses.**

| **Theme** | **Description** |
| --- | --- |
| Opportunities | When respondent listed the need for more opportunities graduate students should participate when taking the lead on leadership tasks |
| Formal programs | When the respondent listed the need for more formalized trainings with some structure (workshops, classes, seminars, etc.) |
| Communication | When the respondent mentioned communication skills (written or verbal) |
| Teamwork | When the respondent referred to the collaboration of others or mentions a group of individuals working together |
| Mentor students | When the respondent commented about the school/faculty encouraging students or monitoring students |
| Mentor undergraduates | When the respondent commented about the students teaching/monitoring undergraduate students |
| Self-development | When the respondent mentioned the need for students to work on their personal skills |
| Business | When the respondent mentioned the need for students to gain knowledge about the business-related skills and disciplines |
| Encouragement | When the respondent mentioned the need for encouraging students to pursue leadership training and/or roles |
| Real world experiences | When the respondent mentioned the new for real world experiences instead of learning in a classroom |
| Education | When the respondent mentioned the need to focus on education |
| Professional | When the respondent mentioned professional development |
| Other | when the respondent mentioned something that wouldn't necessarily fall under any of those categories |
